# Wettability Improvement in Oil–Water Separation by Nano-Pillar ZnO Texturing

**DOI:** 10.3390/nano12050740

**Published:** 2022-02-22

**Authors:** Xiaoyan Liu, Shaotong Feng, Caihua Wang, Dayun Yan, Lei Chen, Bao Wang

**Affiliations:** 1 School of Mechanical Science and Engineering, Northeast Petroleum University, Daqing 163318, China; liu_xydq@163.com (X.L.); fengshaotong0513@163.com (S.F.); wch.dqsy@163.com (C.W.); 2 Department of Mechanical and Aerospace Engineering, The George Washington University, Washington, DC 20052, USA; 3 State Key Laboratory of Tribology, Tsinghua University, Beijing 100084, China; leichen16@mail.tsinghua.edu.cn

**Keywords:** nanostructured surface, wettability, metal fiber felt, oil–water separation, emulsion

## Abstract

The nanostructure-based surface texturing can be used to improve the materials wettability. Regarding oil–water separation, designing a surface with special wettability is as an important approach to improve the separation efficiency. Herein, a ZnO nanostructure was prepared by a two-step process for sol–gel process and crystal growth from the liquid phase to achieve both a superhydrophobicity in oil and a superoleophobic property in water. It is found that the filter material with nanostructures presented an excellent wettability. ZnO-coated stainless-steel metal fiber felt had a static underwater oil contact angle of 151.4° ± 0.8° and an underoil water contact angle of 152.7° ± 0.6°. Furthermore, to achieve water/oil separation, the emulsified impurities in both water-in-oil and oil-in-water emulsion were effectively intercepted. Our filter materials with a small pore (~5 μm diameter) could separate diverse water-in-oil and oil-in-water emulsions with a high efficiency (>98%). Finally, the efficacy of filtering quantity on separation performance was also investigated. Our preliminary results showed that the filtration flux decreased with the collection of emulsified impurities. However, the filtration flux could restore after cleaning and drying, suggesting the recyclable nature of our method. Our nanostructured filter material is a promising candidate for both water-in-oil and oil-in-water separation in industry.

## 1. Introduction

Surface wettability is an important material property, and it is determined by surface structure and surface chemical composition [1,2]. Based on existing theoretical methods, a special material wettability can be achieved by modification of surface texture and surface chemical composition [3,4]. During the last decades, there has been increasing interest in the materials with unique functions due to their promising application in various fields such as self-cleaning, antimicrobial, anti-icing, biocompatible materials, or oil transportation [4,5,6,7,8,9]. Particularly, depending on the different interfacial energy between organic compounds and water in immiscible oil–water mixtures, the materials with special wettability have gained widespread attention in oil–water separation [10,11]. As a simple preparation method, the meshed membrane owned many advantages, such as high permeability of pore space, good oil–water separation efficacy, and promising corrosion resistance [12,13]. Membrane emulsification method attracted plenty of attention. The factors to affect the demulsification performance have been systematically investigated, which included membrane pore size, membrane thickness, transmembrane pressure, as well as emulsion composition [14,15,16,17]. In contrast, traditional methods such as gravity-driven separation, biological treatment, adsorption, and electrochemical techniques have many flaws, such as low separation efficiency, low separation capacity, long time cost, and noticeable secondary pollution [18,19,20].

Furthermore, the structural parameters of filter membranes may play pivotal roles in the performance of oil–water separation, such as pore size, porosity, and membrane thickness [21,22,23]. To date, some simulation and experimental studies have provided clues for filter membrane design. Some representative examples were introduced here. You et al. utilized electrospinning techniques to prepare two fibrous membranes with different nanopore diameters. They found that the physical cutting effect dominated the coalescence process when oil droplets crossed a smaller pore on the membrane [24]. Zhu et al. found that the oil droplets continuously separated emulsion via using squeezing coalescence demulsification (SCD) within a narrow pore size. Their hydrogel nanofiber membranes had a sustained separation capacity. More importantly, these results demonstrated that the pore size, the membrane wettability, and the interfacial tension coefficient of oil–water could control the SCD’s efficiency [25]. Recently, Wei et al. fabricated highly hydrophilic and underwater oleophobic polytetrafluoroethylene membranes, which showed a high oil–water separation efficiency and achieved a high oil–water separation flux. They found that the emulsion separation may be an interfacial problem and may be related to the pore structure of a filtration membrane [26]. Furthermore, Xi et al. manufactured underwater superoleophobic paper-based materials with excellent wet intensity through green papermaking techniques. They also demonstrated that the water flux could be increased by controlling the average membrane pores’ size [27]. These preliminary studies could provide an important basis for the design of filter membranes.

Among these structural parameters, the diameter of the liquid distributor and the hole shape dominated the oil–water separation. To achieve a high demulsification efficiency, the filtering precision should be improved accordingly [28,29]. For the highly precise filtering materials at the submicron scale, a nanostructured membrane is an ideal candidate to improve separation efficacy [30]. In addition, surface energy, a key material characteristic, could only be modified by the coating on that material. Due to the limitation of coating technology, these treatment approaches have been used to promote surface wettability without achieving a satisfied performance in terms of coating combinations [31].

In addition, membrane filtration modified by organic polymer could achieve effective separation of oil–water mixtures. Due to its poor stability in the aqueous medium, however, it is hard to use the common polymer membranes with a special wettability in practice [32]. Therefore, the stable materials such as the inorganic membranes are promising alternatives. To date, all previous studies just focused on the fabrication of hierarchical surface structures without giving adequate attention to the substrate, which determines the material strength and stability.

ZnO nanostructured pillars could be used in complex industrial oil–water mixtures [33,34,35,36]. In this study, the stable ZnO nano-pillars were prepared on a high-intensity metal fiber filter by crystal growth, which had a good combination with the substrate. Therefore, the modified filter material possessed stability of mineral coating and strength. Our method promoted the surface wettability and achieved both a superhydrophobicity in oil and a superoleophobic property in water. The filter membrane modified by nano-pillars could be stably used in both water-in-oil and oil-in-water conditions. The filtration flux variation with the collection of emulsified impurities was also investigated. This study provided a novel strategy to achieve stability and strength of a mineral coating simultaneously and might have wide application in separating water-in-oil and oil-in-water emulsions.

## 2. Materials and Methods

### 2.1. Preparation of ZnO Seed Layers

The stainless-steel fiber felts with the filtration precision of 5 µm were used as a substrate in this study. To eliminate surface contamination, all substrate samples were ultrasonically cleaned with absolute ethanol and ultra-pure water for 10 min. (CH3COO)_2_Zn (0.75 M (CH3COO)_2_Zn) (Sinopharm Chemical Reagent Co., Ltd., Shanghai, China) and Monoethanolamine ((CH3COO)_2_Zn and Monoethanolamine in the proportion 1:1) (Aladdin Biochemical Technology Co., Ltd., Shanghai, China) were dissolved in a 2-methoxyethanol solution (Aladdin Biochemical Technology Co., Ltd., Shanghai, China). This solution was magnetically stirred and heated in a temperature-controlled water bath (70 °C, 60 min). The seed solutions were obtained by standing at room temperature for 24 h. Substrates are submerged in the seed solution and lifted repeatedly. Finally, the substrates with the ZnO nanostructured coating were sintered (350 °C, 20 min) to stabilize the coating.

### 2.2. Preparation of ZnO Nano-Pillar Coated Substrates

**Figure 1:**
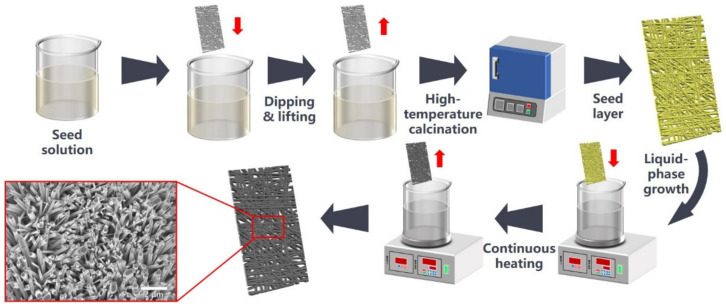
Schematic illustration of manufacturing ZnO modified metal fiber felt.

As shown in Figure 1, the seed layer coated substrates were first immersed in Zn(NO_3_)_2_ (Sinopharm Chemical Reagent Co., Ltd., Shanghai, China)/hexamethylenetetramine (HMTA) (Sinopharm Chemical Reagent Co., Ltd., Shanghai, China)/DAP (Macklin Inc., Shanghai, China) (0.05 M Zn(NO_3_)_2_, Zn(NO_3_)_2,_ and DAP in the proportion 1:6) aqueous solution (10 min) and then was heated in a constant temperature water bath (90 °C, 3 h). After the reaction, the substrates were separated from the solution and washed with ultra-pure water to obtain the substrates with ZnO nanostructures.

### 2.3. Characteristic Analysis

A field emission scanning electron microscope was used to examine the surface morphology of the samples as they were developed (SEM, HITACHI-SU8220, Hitachi, Ltd., Tokyo, Japan). Chemical component was characterized by energy dispersive spectrometry (EDS, Bruker, Billerica, MA, USA). Data-physics was used to characterize surface wettability, including water contact angle (WCA), oil contact angle (OCA), underoil–water contact angle (UWCA), and underwater–oil contact angle (UOCA) (Data-Physics, DataPhysics Instrumente GmbH., Filderstadt, Germany). The liquid droplets utilized for WCA, UWCA, CA and UOCA analyses had a volume of 4 μL. The amount of water remained in the collected filtrates was determined by Karl Fischer Titrator (TP653, TimePower Measure and Control Equipment Co. Ltd., Beijing, China). Total organic carbon (TOC) equipment was used to measure the oil content of the filtrates (TOC-L, Shimadzu (Shanghai) Global Laboratory Consumables Co. Ltd., Shanghai, China). The Zetasizer Nano ZS (Malvern Instruments Ltd., Malvern, UK) was used to investigate the dispersion of water droplets in oil and oil droplets in water. The microscopy pictures of W/O and O/W emulsions were acquired by using an inverted microscope (Keyence Corporation, Osaka, Japan).

### 2.4. Oil–Water Separation

**Figure 2:**
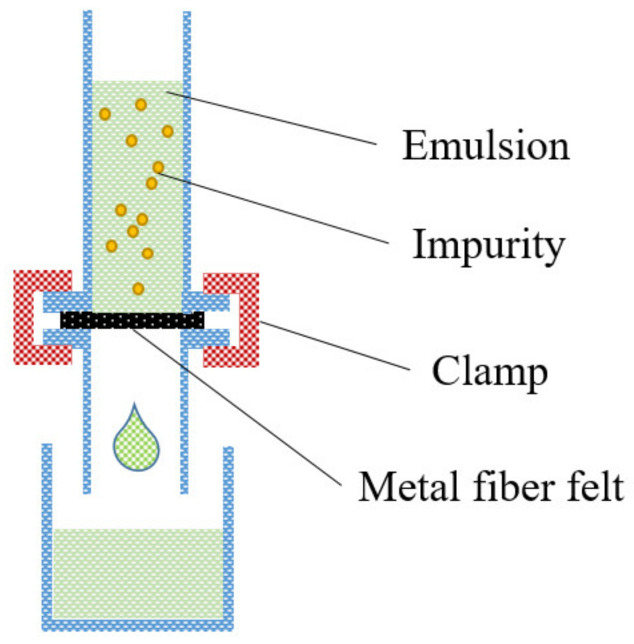
Schematic diagram of experimental setup.

Here, 99.6 g of infused oils (Diesel, Decane, Dodecane, and N-tetradecane) were combined with 0.4 g of water and ultrasonically agitated for 1 min to create water-in-oil(W/O) emulsions. To prepare oil-in-water (O/W) emulsions, 0.4 g of oil (Diesel, Decane, Dodecane, and N-tetradecane) was combined with 99.6 g of ultra-pure water and ultrasonically agitated for 10 min. The experiment of separation was performed by using a home-made filter device that sandwiched the created membrane between two glass tubes. Before assembling the separation device, the prepared filter membranes were placed in ultrapure water or oil solutions for 30 s, dependent on the types of emulsions. The pre-wetted filter membranes were used in the separation experiments. A glass cylinder tube containing metal fiber felt as a filter was placed at the position shown in Figure 2 to realize filtration of oil–water emulsions under gravity. The filtrate was collected at the bottom of the vessel after the emulsion was poured through the open end of the glass cylinder. The oil–water emulsion was quickly poured into the home-made separation device. The liquid column was sustained at a depth of 10 cm during the separation. The separation efficiency was estimated by the following equation:

(1) $$\eta =1-\frac{C_{1}}{C_{0}}$$

where *C*_0_ represented the dispersed phase content in the initial W/O and O/W emulsions (ppm); *C*_1_ represented the water/oil content in the collected filtrate (ppm); and *η* represented the separation efficiency. Meanwhile, the filtration flux (*L*) was calculated by:

(2) $$L=\frac{m}{\rho \pi r^{2}t}$$

where *m* was the mass of collected filtrate measured with a balance scale; *ρ* was the density of water/oil (*ρ* = 10^3^ kg/m^3^); *r* was the radius of a glass tube (the radius of the glass tube in this study was 8 mm); *t* was the filtration duration, with the filtration flux computed by measuring the quantity of filtrate 1 min per time.

## 3. Results and Discussion

### 3.1. Surface Morphology and Chemical Composition of ZnO Coated Metal Fiber Felt

The seed layer was coated on a metal fiber felt substrate using the sol-gel method. We fabricated ZnO-coated metal fiber felts by immersing the metal fiber felts with the seed layer into the precursor solution for the liquid phase growth process (Figure 1). At the initial growth stage, HMTA tardily decomposed to liberate OH^−^ ions. The generated OH^−^ ions form insoluble ZnO precipitated at supersaturated Zn(OH)_4_^2−^ ions or Zn^2+^ ions. As a polar crystal, ZnO (0001) exhibited a positive charge on the crystalline surface and attracted OH^−^ ions and Zn(OH)_4_^2−^ ions in the solution. As a result, ZnO had the highest growth rate along the C-axis compared to other crystal planes and eventually forms ZnO nanostructures [37,38,39,40]. The overall chemical reactions were presented as follows:

C_6_H_12_N_4_ + 6H_2_O → 6HCHO + 4NH_3_

NH_3_ + H_2_O → NH_4^+^_ + OH^−^

Zn^2+^ + 2OH^−^ → ZnO(s) + H_2_O

**Figure 3:**
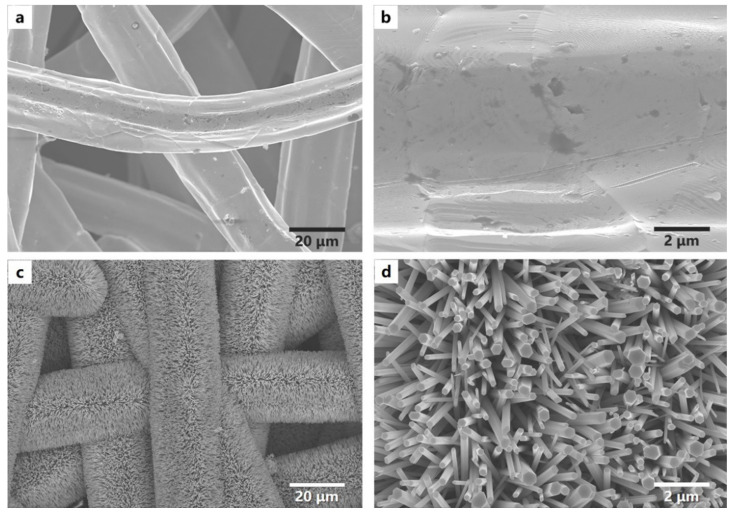
SEM images of original filter membrane (**a**,**b**). SEM images of ZnO-coated filter membrane (**c**,**d**).

SEM images indicated that the raw stainless-steel fiber felt was composed of metal fibers with an intricate arrangement and the surface of the metal fibers was smooth (Figure 3a,b). The average metal fiber diameter of the fabricated substrates increased, and the average pore size decreased after being coated with ZnO nanostructures (Figure 3c,d). The zoomed-in imaging showed that ZnO had nano pillar-like structures. Furthermore, the ZnO nanopillars in our growth experiments had hexagonal cross section with an average top diameter of 185 ± 81 nm. Finally, ZnO nanostructures wrapped metal fibers’ surface without blocking pores.

**Figure 4:**
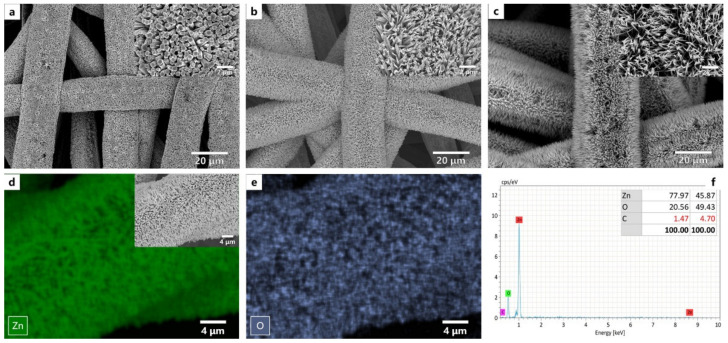
SEM imaging of ZnO-coated filter membranes manufactured with various growth solution concentrations: (**a**) 0.025 mol/L (MF-0.025); (**b**) 0.05 mol/L (MF-0.05); (**c**) 0.1 mol/L (MF-0.1) (The length of the white scalebar in (**a**–**c**) is 20 μm, and the size of scalebar in the zoomed picture is 2 μm); (**d**,**e**) the original EDS mapping of Zn and O elements in a ZnO coated filter membrane; (**f**) EDS elemental analysis.

The liquid-phase ZnO growth had two stages: dissolution and crystallization. The growth solution concentration was one of the critical parameters for ZnO’s nucleation and growth. The effect of growth solution concentration on the formation of ZnO coating on filter membrane was shown in Figure 4. We found that the surface was accumulated by dense ZnO structures at low growth solution concentrations (Figure 4a). The nanostructures had a slight pitch and low ratio of length to neck diameter. During crystal growth, the concentration of growth solution determined the solutions’ concentration at the center of crystal growth face. As the concentration of growth solution increased, the supersaturation of Zn-ions in the solution was enhanced. It led to apparent orientation growth behavior and eventually formed nanoarray structures (Figure 4b). When the concentration increased to 0.05 mol/L, the filter membrane surface presented arranged pillar-like structures. A large gap between nanostructures facilitated the droplets’ immersion into nanostructures. The average diameter of nano-pillars decreased from 314 ± 52 nm to 91 ± 24 nm, and the average length of the nano-pillars just increased slightly. ZnO nanostructure transformed from nano-pillars to nano-needles (Figure 4c). The decrease in the diameter of the nanostructures increased the size of structural interstices, resulting in a coating deficiency on metal fibers and weakened stability of ZnO coatings. Energy-dispersive X-ray spectroscopy (EDS) data were shown in Figure 4d–f. Zn and O element distribution and measurements in coated metal fiber confirmed that the final coating was constituted of ZnO with an excellent coverage density.

### 3.2. Surface Wettability

**Figure 5:**
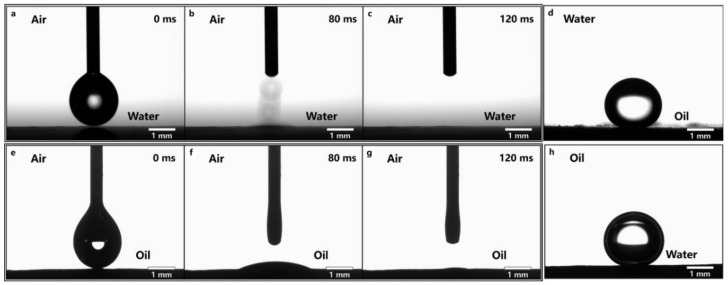
(**a**–**c**) Imaging of a water droplet spreading through coated filter membrane; (**d**) imaging of an underwater oil droplet on a filter membrane with a 151.4° contact angle; (**e**–**g**) imaging of oil droplets spreading through coated filter membrane; (**h**) imaging of an underoil water droplet on a filter membrane with a 152.7° contact angle.

The contact angle measurements were accustomed to analyzing the surface wettability of as-prepared filter membranes, as illustrated in Figure 5. Additionally, the wettability of the studied surface layers was evaluated using MF-0.05 as a test sample. A droplet of water was dropped onto a ZnO-coated filter membrane in the air. It diffused rapidly within 80 ms, which suggested that the coated filter membrane had satisfactory hydrophilicity (Figure 5a–c). Furthermore, the oil droplet swiftly spread on the as-prepared filter membrane in the air, demonstrating a superoleophilicity (Figure 5e–g). However, the oil droplet could not spread on the as-prepared filter membrane in the water, illustrating that the coated filter membrane had an excellent oil repellency once submerged (Figure 5d). A drop of water was also unable to spread on the filter membrane previously prepared, which demonstrated the filter membrane’s outstanding repellence towards water underoil (Figure 5h). ZnO coated filter membrane had a static underwater oil CA of 151.4° ± 0.8° and underoil water CA of 152.7° ± 0.6°, respectively, exhibiting both superhydrophobicity in oil and superoleophobic properties in water.

### 3.3. Separation of Oil-in-Water Emulsions

**Figure 6:**
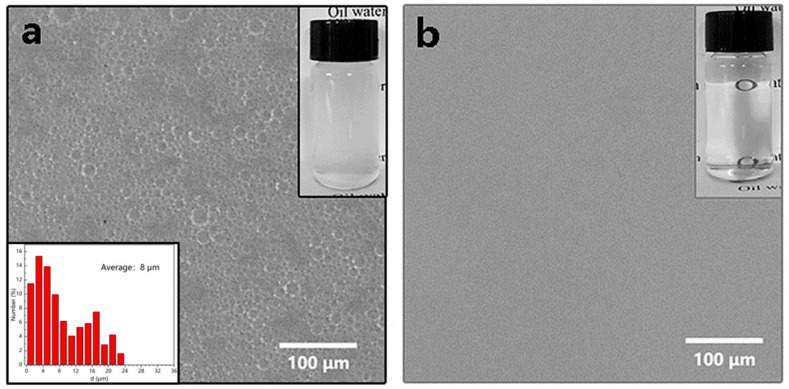
The microscopic and optical images of O/W emulsions (**a**) before separation and average size of oil droplets; (**b**) filtered with filter membrane coated with ZnO.

In contrast to a stratified oil–water mixture, an oil-in-water emulsion containing emulsified oil droplets with various sizes was difficult to be separated. To prevent emulsified droplet penetration and achieve separation of O/W emulsions, the micropore size was one of the important characteristics of separation membranes. Here, the capacity of ZnO coated filter membrane to separate in the O/W emulsion was demonstrated. To analyze the separation efficacy of an as-prepared filter membrane, emulsions of diesel/water, N-decane/water, dodecane/water, and N-tetradecane/water were invented. Figure 2 illustrated that the apparatus utilized separate O/W emulsions. The cleaned water was gravity-fed through the membrane and collected in the beaker underneath, while the as-prepared emulsions were poured into the top tube. The electronic balance recorded the mass of the collected filtrate in real-time. The separation findings of dodecane/water emulsion were illustrated in Figure 6. As shown in the microscopic imaging, the prepared water/dodecane emulsion contained a large amount of micron-sized oil droplets with an average size distribution of 8 μm. The collected water was transparent after filtration across the as-prepared filter membrane and there were no oil droplets revealed when observed under an inverted microscope.

**Figure 7:**
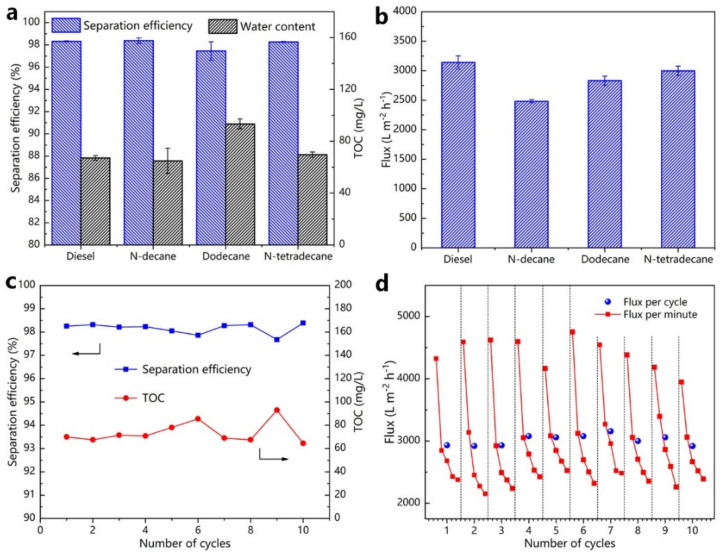
(**a**) The separation efficiency and TOC in the filtrates of oil-in-water emulsions; (**b**) various oil-in-water emulsion separation fluxes; (**c**) the recycling separation of the as-prepared filter membrane for O/W emulsions; (**d**) separation flux stability in 10-cycle experiments.

Furthermore, other emulsions such as diesel/water, N-decane/water, and N-tetradecane/water were successfully separated (Figure 7a,b). As expected, the as-prepared filter membrane successfully separated the four emulsions, but only left minimal residual oil in the filtrate. All the oil-in-water emulsions exhibited the promising permeation fluxes. The highest flux of 3139 L·m^−2^·h^−1^ was obtained for diesel-in-water emulsion. These findings suggested that the ZnO-coated filter membrane could separate a variety of O/W emulsions. Though the separation efficiency obtained in this study was similar with previous studies using inorganic coating materials (Nano-ZnO and Nano-TiO_2_) to separate oil-in-water emulsions, we obtained a higher separation flux performance than these studies [41,42,43]. In addition, the as-prepared filter membrane was evaluated by a ten-times recycling separation of the dodecane/water emulsion to indicate the characteristic of reusability. The membrane was ultrasonic-assisted cleaned with absolute ethyl alcohol for 5 min after each cycle of the experiment (collected filtrate volume was 80 mL), and it was immediately used for the following filtration test. After 10 recycles, the TOC concentration was less than 72 mg/L and the separation efficiency remained higher than 98%, confirming its capability for standing oil–water separation application (Figure 7c). Moreover, our results demonstrated an excellent cyclic stability of the filter membrane coated with ZnO, although the variations of flux in each cycle decreased with the increase in filtration duration (Figure 7d).

### 3.4. Separation of Water-in-Oil Emulsions

**Figure 8:**
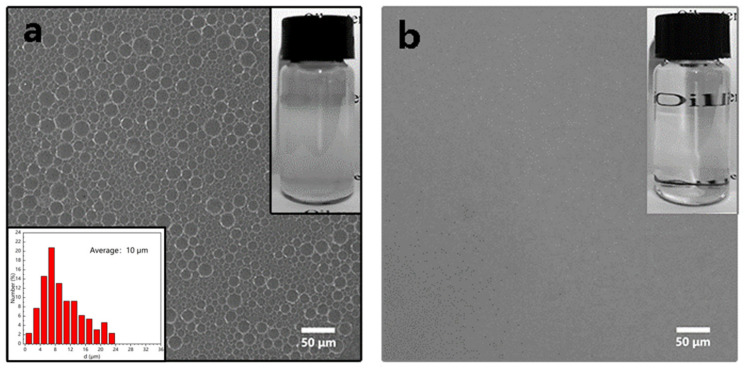
The microscopic and optical images of surfactant-stabilized water-in-oil emulsions (**a**) before separation average size of water droplets; (**b**) filtered with filter membrane coated with ZnO.

The ZnO-coated filter membrane could separate O/W emulsion and O/W emulsion. The water content in the filtrate, separation flux, and corresponding separation efficiency were used to analyze the separation performance of the as-prepared filter membrane. As demonstrated in Figure 8a, the appearance of the W/O emulsion was cloudy and contained sufficient micron-sized water droplets with an approximate average size of 10 μm. There were no water droplets in the liquid filtered by the ZnO-coated filter membrane (Figure 8b).

**Figure 9:**
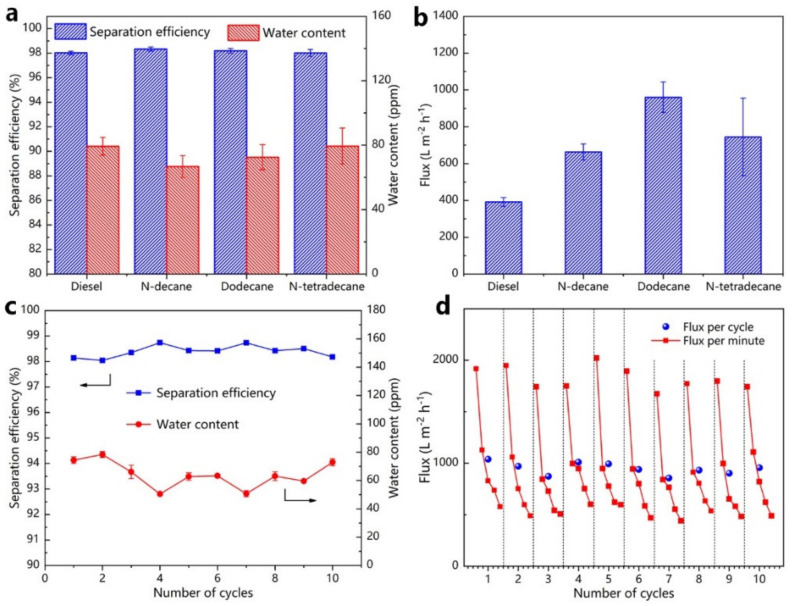
The separation efficiency and water content in (**a**) water-in-oil emulsions filtrates; (**b**) filtration fluxes of various water-in-oil emulsions; (**c**,**d**) the recycling separation performance of ZnO coated filter membrane for water-in-oil emulsions.

Figure 9a demonstrated that the as-prepared filter membrane was capable of accurately separating various W/O emulsions, such as water/diesel, water/N-decane, water/dodecane, and water/N-tetradecane. The efficiency of separation of the as-prepared filter membrane for various water-in-oil emulsions were more than 98%. Furthermore, the water concentration for the filtrates of different emulsions was less than 80 ppm. The following Equation (2) was used to estimate the filtration flux of the as-prepared filter membrane, and fluxes (L) for different emulsions were evaluated for each minute. The separation fluxes for water/diesel, water/N-decane, water/dodecane, and water/N-tetradecane emulsions were about 392, 663, 960, and 744 L·m^−2^·h^−1^, respectively (Figure 9b). The separation efficiency obtained in this study was similar to existing studies about inorganic coating materials, which were used to separate water-in-oil emulsions. However, the separation flux obtained here was lower than in all these studies [44,45].

As an example, the reusable separation capability of the as-prepared filter membrane was accessed by the separation experiment of water/N-decane. The separation of each cycle lasted for 8 min, followed by a 5 min ultrasonically cleaned with absolute ethyl alcohol. Figure 9c showed that even after 10 cycles of use, the efficiency of separation of the as-prepared filter membrane was always greater than 98%, and the water content in the filtrates was lower than 80 ppm, suggesting that the as-prepared filter membrane was more recyclable. Furthermore, during the reusability experiment, the filtration flux did not change appreciably, suggesting the filter membrane had excellent separation efficiency and splendid stability (Figure 9d).

### 3.5. Demulsification Mechanism in Oil-in-Water and Water-in-Oil Emulsion

**Figure 10:**
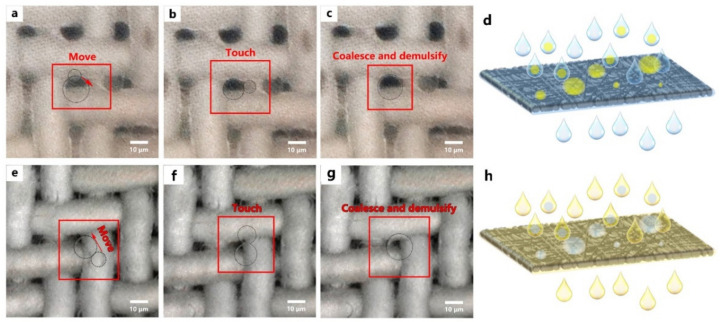
Mechanism of demulsification process of oil-in-water and water-in-oil emulsions; (**a**–**c**) coalescence and demulsification of emulsified oil droplets; (**e**–**g**) coalescence and demulsification of emulsified water droplets; (**d**,**h**) suppositional separation process of oil-in-water and water-in-oil emulsions.

ZnO nanostructures had achieved both a superhydrophobicity in oil and a superoleophobic property in water. As shown in Figure 10a–c, the separation mechanism could be concluded as follows: it was due to the excellently amphiphilic property in air; the as-prepared filter membrane was extensively wetted by the water phase when it interacted with an oil-in-water emulsion, allowing the water phase to easily infiltrate through. The emulsified oil droplets were then intercepted and adsorbed on the filter membrane in the second step. Finally, oil droplets attracted and coalesced with other emulsified droplets, allowing oil-in-water emulsions to be separated efficiently. Moreover, these water droplets captured by the membrane during the separation process were rapidly coalesced and demulsified to create bigger droplets (Figure 10e–g).

**Figure 11:**
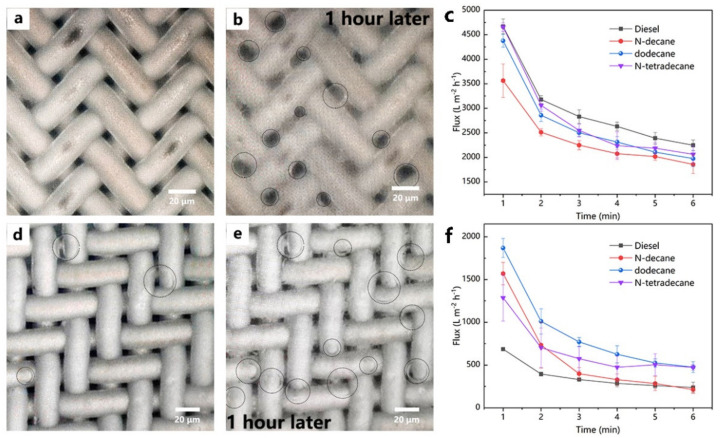
Image of metal fiber felt surface in the separation process of (**a**) oil-in-water emulsions and (**d**) water-in-oil emulsions; (**b**,**e**) the performance oil–water separation through one hour; (**c**,**f**) variation of separation fluxes with each minute.

Despite the above results and analysis, the flux change in separation process deserves further study. The oil droplets constantly agglomerated and adsorbed to the fibers during oil-in-water emulsion separation. As illustrated in Figure 11a,b, the oil phase was intercepted to access the filter membrane’s surface. The oil–water flux through the membrane was determined by membrane porosity, pore size, as well as pore distribution. Current study found that relatively large pore size leads to a high separation flux. Some parts of porous structures would be blocked by the absorbed oil droplets on the membrane, which resulted in a decreased porosity and a shortened effective separation area of the membrane. In other words, the oil–water separation was saturated with the duration, which significantly decreased the oil–water separation flux. As envisioned, our results suggest that the variations of permeation flux decreased with the increase in filtration duration. The flux decline was in the range of 45–55% (Figure 11c). The same trend was observed during the separation of a water-in-oil emulsion (Figure 11d–f). In addition, the fluxes in separation process varied with the time (Figure 11c,f). As the accumulation of droplets increased on the surface, the pore size decreased and eventually changed the influx during the emulsion separation process. Our observations were consistent with the previous studies with similar focuses. For example, the separation flux of membranes with different pore sizes was reported at a range between 250 and 4300 L·m^−2^·h^−1^ [15,41]. We need to point out that the data obtained from a single moment may not accurately describe the flux change in the separation process. It will be more practical if we focus on a general description of the entire separation process or the average data shown in Figure 7d and Figure 9d. Finally, it was needed to point out that the filter membrane could continue to adsorb emulsified droplets even after a simple cleaning.

## 4. Conclusions

Herein, the surfaces with special wettability were achieved by ZnO nanostructures, which could improve oil/water separation efficiency. In this study, ZnO nanostructures were fabricated by a two-step process for the sol-gel process and crystal growth from the liquid phase to achieve both a superhydrophobicity in oil and a superoleophobic property in water. Our study demonstrated an excellent wettability of nanostructured stainless-steel fiber felt. The ZnO coated filter membrane had a static underwater oil contact angle of 151.4° ± 0.8° and underoil water contact angle of 152.7° ± 0.6°, respectively.

In oil–water separation, the emulsified impurities in both water-in-oil and oil-in-water emulsion liquid were effectively intercepted to achieve an oil–water separation. The filter material with small pores (~5 μm diameter) could separate diverse water-in-oil and oil-in-water emulsions with a separation efficiency higher than 98%. Based on the oil–water separation mechanism, we could predict that the blocking of filter materials was caused by the collection of emulsified impurities. Thus, the effect of filtering quantity on the separation efficacy was investigated to analyze the influence of impurities. The investigation results showed that the filtration flux decreased with the collection of emulsified impurities. However, the filtration flux could restore after cleaning and drying, suggesting the recyclable nature of our strategy. The nanostructured filter material is a promising candidate for separating both water-in-oil and oil-in-water emulsion in industry. The proposed filter materials in this study may be repetitively used without any treatments, secondary pollution, and low energy consumption. Therefore, our methods may have wide application in the future.

## Data Availability

The data presented in this study that support the findings are available on reasonable request from the corresponding author.
